# The Association Between Inflammatory Markers and Hypertension. A Call for Anti-Inflammatory Strategies?

**DOI:** 10.1100/tsw.2006.190

**Published:** 2006-10-09

**Authors:** Néstor H. García, Luis I. Juncos

**Affiliations:** J. Robert Cade Foundation CONICET, Córdoba, Argentina

**Keywords:** hypertension, kidney, angiotensin II, nitric oxide, inflammation, tubulointerstitial inflammation, statins

## Abstract

The most important goal of antihypertensive therapy is to prevent the complications associated with hypertension (stroke, myocardial infarction, end-stage renal disease, etc). For this, secondary targets such as left ventricular hypertrophy, proteinuria, dementia, and other signs of hypertension-induced organ damage help the physician to assess risks and monitor treatment efficacy. New treatment targets may be arising, however. One such target may be endothelial dysfunction. In effect, endothelial dysfunction not only may precede the elevation of blood pressure, but may also pave the way to conditions often associated with hypertension, such as diabetes, arteriosclerosis, microalbuminuria, congestive heart failure, and tissue hypertrophy. Because inflammation often accompanies endothelial dysfunction, approaches to counteract inflammation are now being evaluated. For this, antagonists of the renin-angiotensin-aldosterone system, statins, and beta blockers are all being tested. All of these agents seem to prevent or delay the induction of proinflammatory molecules aside from, and in addition to, their specific effects on blood pressure. The focus of this review is to update some of the animal and human research showing that hypertension sets off an inflammatory state and also to consider some of the anti-inflammatory approaches that may prevent the development of endothelial dysfunction, and the subsequent renal and cardiovascular damage.

## INTRODUCTION

Despite extensive basic and clinical investigation in the field of hypertension, high blood pressure remains the most important cause of cardiovascular death in developed countries. This lack of success reveals the failure of our current approach in preventing untimely death and suggests that we may be missing critical therapeutic targets. Such an ominous outlook may change, though, as new approaches aim toward earlier disease mechanisms. In this respect, the recently recognized arterial wall inflammation, present early in the course of hypertension, may prove to be a more specific and effective target for treatment[1,2].

Indeed, chronic low-grade arterial inflammation and its resultant endothelial dysfunction have emerged as potential mechanisms underlying the progression of hypertension and arteriosclerosis[3,4], thus triggering a number of studies to assess the role of inflammatory markers in these conditions.

One such marker is the high-sensitivity C-reactive protein (CRP). CRP, the most studied inflammatory marker, may in fact play a direct pathogenic role in the development of the atherosclerotic lesion[4]. In effect, in the absence of active infection, measuring CRP has become a reasonable alternative in the assessment of arterial wall inflammation, not only because it is highly replicable, but also because it is a risk factor comparable to total serum cholesterol measurement[5]. In this respect, CRP appears as a risk factor more potent than tumor necrosis factor-α, interleukin-6, *iso*-PGF2α, or serum amyloid. Supporting this notion, a number of therapeutic agents currently available, such as aspirin, statins, angiotensin-converting enzyme inhibitors (ACE-I), thienopyridines, and peroxisome proliferator-activated receptor agonists, have been shown to reduce CRP and arterial inflammation. Unfortunately, although it is clear that elevated CRP denotes increased risk and emerging evidence suggests the merit of novel therapies that lower CRP, it remains unanswered whether suppression of inflammation and the consequent lowering of CRP translate into decreased clinical events. For this, it is essential to understand the mechanisms leading to inflammation in hypertension.

## EVIDENCE OF INFLAMMATION IN HYPERTENSIVE MODELS

The first evidence relating an immune-inflammatory mechanism to the development of hypertension was advanced by Svendsen[6]. This investigator showed that an intact thymus is necessary in the DOCA-salt model of hypertension. In effect, lymphocytes displaying delayed-type immune reactivity infiltrate the perivascular interstitium in the kidney. Other evidence came from studies showing that cyclophosphamide therapy, antithymocyte serum, neonatal thymectomy, and thymic implants from normotensive donors can ameliorate hypertension in various rat models. These early results were considered as evidence that hypertension could result from autoimmune vasculitis. In fact, the immune dysfunction observed in spontaneously hypertensive rats (SHR) was considered to be an adaptive defense mechanism against otherwise life-threatening hypertension[7]. In contrast, a recent report has provided evidence that immune cells accumulating in the kidney may mediate sodium retention and by this means may increase blood pressure[8].

This comes as no surprise, because tubulointerstitial infiltration by lymphocytes and macrophages seems to be present consistently in experimental models of salt-sensitive hypertension (Table 1).

**TABLE 1**
**Models of Salt-Sensitive Hypertension Associated with Tubulointerstitial Infiltration**

| |
|---|
| DOCA-salt hypertension |
| Postangiotensin II–infusion salt-sensitive hypertension |
| Postcathecholamine-infusion salt-sensitive hypertension |
| Hyperuricemia-induced salt sensitivity |
| Hypertension after chronic nitric oxide synthesis inhibition |
| Hypertension associated with protein overload proteinuria |
| Two-kidney one-clip hypertension |
| Aging nephropathy |
| Cyclosporine nephropathy |
| SHR |
| Stroke-prone SHR |

Moreover, several investigators have shown a correlation between the number of infiltrating cells and the severity of hypertension[7]. Finally, strategies to decrease renal inflammatory cell infiltrates also prevent the development of salt-sensitive hypertension[9,10] and improve established hypertension in genetically prone strains of hypertensive rats[11].

The mechanisms activating this inflammatory process are yet to be elucidated. Nevertheless, there is agreement supporting the notion that sodium retention, vascular remodeling, smooth muscle tone regulation, and modulation of inflammation are maintained by the balance between nitric oxide (NO), angiotensin II (Ang II), endothelin-1 (ET-1), cytokines, and the vascular generation of reactive oxygen species (ROS)[12,13,14].

Launching this inflammatory process requires the engagement of circulating cells and for this, the endothelium expresses a number of adhesion molecules. High blood pressure, high serum cholesterol, oxidized LDL, and Ang II increase the synthesis of adhesion proteins, such as LTB4, LFA-1, P-Selectin, ICAM-1, IL-6, IL-1β, MCP-1, PAF, and others (MCP-1 is increased also by aging[15].) All these molecules stimulate leukocyte-endothelial cell adhesion and migration to the vascular tissue or to the renal interstitium[16]. NO also takes part in the inflammatory response. In effect, endothelial nitric oxide synthase (eNOS) increases the synthesis of TGF-β1[17] and MCP-1[18].

It follows then that in all these processes, Ang II and NO may interact to induce the inflammatory response. In fact, Ang II not only regulates NO production and eNOS expression, but also impairs NO activity by increasing oxidative stress through the stimulation of NADH/NADPH oxidase in vascular smooth muscle cells and endothelial cells. Conversely, NO down-regulates the AT1 receptor[17]. Treatment of rat vascular smooth muscle cell with NO donors inhibits Ang II binding to cells, without altering receptor affinity. However, treatment of the cells with cGMP analogs has no significant effect on Ang II binding, suggesting that NO regulates Ang II receptors through a cGMP-independent mechanism. In vascular smooth muscle cells, inhibition of Ang II binding by NO is caused by decreased AT1R mRNA expression and occurs at the transcriptional level[17]. In addition, downstream effectors of Ang II and NO signaling pathways also interact with each other. This explains, then, the continuous interplay of Ang II and NO in the maintenance of interstitial integrity.

The interactions of these two vasoactive systems take place in infiltrating cells and in vascular tissue, amplifying both inflammation and hypertension. Interestingly, some observations indicate that inflammation by itself could increase blood pressure during salt loading. Indeed, Shifra et al[18] showed that SBH/y rats on normal sodium diet exhibit high rates of superoxide release, increased circulating PMNL count, increased carbonyl fibrinogen levels and MDA, and decreased GSH serum levels. Despite the presence of all of these markers of inflammation, blood pressure remains normal. When these rats are switched to a high salt intake, blood pressure increases. Thus, at least in this model, the inflammatory process precedes the development of hypertension.

## EVIDENCE OF INFLAMMATION IN HUMAN HYPERTENSION

Experimental data showing the role of inflammation in human hypertension is limited compared to the data in atherosclerosis, acute coronary syndrome, and heart failure. Nonetheless, it is well accepted that the multifactorial nature of hypertension is attended by low-grade inflammation. In support of this notion, a number of studies have shown a relationship between inflammatory markers and hypertension. Perhaps the first of these studies to show an association between inflammatory markers and blood pressure levels was done by Chae et al.[12]. These authors tested the hypothesis that hypertension may increase the risk of atherosclerosis via proinflammatory effects. For this, they did a cross-sectional study in 508 apparently healthy men, assessing the association between blood pressure and baseline plasma concentrations of intercellular adhesion molecule-1 (ICAM-1) and interleukin-6 (IL-6), two markers of inflammation. Increased systolic blood pressure, pulse pressure, and mean arterial pressure were associated with higher levels of ICAM-1. In addition, diastolic blood pressure was significantly correlated with IL-6 levels. This association between ICAM-1 and IL-6 with hypertension suggested that inflammation may promote the development of atherosclerotic disease in the hypertensive.

Subsequently, the Women's Health Study investigated whether highly sensitive CRP concentrations correlated with the development of hypertension[19]. This was a prospective study of 20,525 female U.S. health professionals, aged 45 years or older, followed for up to 7 years. Indeed, CRP was associated with an increased risk of developing hypertension in all evaluated groups, including those with very low levels of baseline blood pressure. The association was seen also with no traditional coronary risk factors. Even though these associations were apparent with low baseline blood pressure levels, no adjustment could be made for the presence of the metabolic syndrome, a possible mediator of this connection.

Likewise, Niskanen et al.[20] investigated the role of inflammation in the development of hypertension. For this, they studied the association of highly sensitive CRP concentration, smoking, and abdominal obesity with the appearance of hypertension in men. Prehypertensive individuals were followed for 11 years. Men with CRP > 3.0 mg/l were 2.8 times more likely to develop hypertension than men with CRP < 1.0 mg/l, even after adjustment for features of the metabolic syndrome, lifestyle factors, and their changes. Moreover, CRP correlated positively with heart rate and elevated heart rate predicts a greater risk of developing hypertension[21]. In brief, this study suggests that inflammation precedes the development of hypertension. On the other hand, hypertension may be a cause rather than a consequence of inflammation. This is based on studies showing that various antihypertensive treatments lower the serum levels of inflammatory markers (see below).

Be that as it may, the increased expression of various inflammatory markers may proceed afterward through a common path. A good example is pre-eclampsia, a rather unique form of hypertension during pregnancy. In this condition, the endothelial synthesis of MCP-1 increases 1.5-fold whereas interleukin-8 (IL-8) raises 2.5-fold, compared to values in normal pregnancy. These changes are associated with increased oxidative stress, increased circulating levels of oxygen-free radicals, and a 4.5-fold rise in lipid peroxides[22]. Conversely, circulating antioxidants are decreased. All of these changes are attended by endothelial cell dysfunction.

Other leukocyte-derived inflammatory markers have been reported to be elevated in human hypertension, indicating that multiple oxidative mechanisms take place in hypertension[23]. In effect, increased levels of 12-LO [leukocyte-type 12/15-lipoxygenase (LO)] and its product, 12-hydroperoxyeicosatetraenoic acid [12-(S)-HETE], have been observed in patients with essential hypertension[24]. In hypertensive patients, lowering blood pressure with Ang II receptor blockers (AIIRB) is accompanied by diminished levels of malondialdehyde (MDA), a marker of oxidative stress[25], and by reversion of hypertension-induced structural changes in resistance arteries[26]. Taken together, these observations strongly suggest that oxidative stress plays an important role in the pathogenesis of hypertension.

## INTERVENTIONS TO PREVENT INFLAMMATION

In the last few years, a number of studies have shown that drug treatment renders beneficial effects that are independent from their antihypertensive or specific metabolic actions. For instance, the renin-angiotensin system (RAS) antagonists lower serum and tissue levels of inflammatory markers at least in part by reducing angiotensin-induced oxidative stress. Other agents, such as the hydroxy-methyl glutaryl Co A reductase inhibitors (statins) that may lower blood pressure may**,** in addition**,** diminish tissue inflammation by other mechanisms. Thus, treatment directed to block the RAS or cholesterol pathways may be of help in preventing inflammation and disease progression. Similarly, aldosterone and the sympathetic system exert profibrotic effects that may be blocked by specific antagonists, regardless of their antihypertensive actions.

## Blocking the Renin-Angiotensin System

Experimental models have shown that Ang II plays an important role in renal and cardiovascular disease progression, particularly by promoting inflammation, tissue hypertrophy, interstitial fibrosis, and other changes. Thus, interventions to inhibit the RAS are expected to prevent these harmful effects. In effect, ACE-I and AIIRB have been shown to decrease tissue fibrosis and end organ damage in animal and human studies. Because the efficacy of organ protection differs with various drug classes, mechanisms other than decreased blood pressure have been suspected. In this respect, inflammation is a major suspect because of its relationship to the RAS.

A good model of angiotensin-induced tissue inflammation is malignant hypertension. Indeed, this condition portrays a striking activation of the RAS and severe vascular changes. In the laboratory, malignant hypertension can be induced in transgenic rat models (characterized by overexpression of genes of the RAS)[27] and in the 2-kidney, 1-clip Goldblatt model (comparable to human renovascular hypertension). These two models support the notion that Ang II plays a most important role in the development of malignant hypertension. In both, interventions to inhibit the RAS[28] prevented the development of severe vascular damage even though high blood pressure remained uncontrolled. The absence of histologic features of malignant hypertension was accompanied by lower expression of MCP-1 and decreased interstitial influx of macrophages[29]. In brief, these findings suggest that Ang II plays a role in the inflammation of malignant hypertension.

In fact, Ang II mediates a wide variety of inflammatory processes. For instance, Ang II activates the proinflammatory transcription of nuclear factor kappa B (NF-κB) in human monocytes, human vascular smooth muscle cells, and human endothelial cells. Angiotensin also activates NF-κB, leads to increased production of vascular cell adhesion molecule-1[30], MCP-1[31], and IL-6[32], all of which are involved in the development of tissue inflammation not only in the kidney, but also the cardiovascular system. All of these mediators are inhibited by Ang II blockade.

Remarkably, the proinflammatory role of Ang II goes beyond the mere induction of hypertension as shown in normotensive, hypercholesterolemic models. Indeed, in a nonhuman primate model of atherosclerosis, ACE-I and AIIRB significantly reduced the progression of atherosclerosis without reducing blood pressure and plasma cholesterol levels[33].

Clinical studies validate these results. In effect, Dahlöf et al.[33] have reported in humans that ACE-I reverse left ventricular hypertrophy (LVH) better than calcium channel blockers and beta blockers. In support of this notion, Diez et al. have reported diminished collagen synthesis in myocardium of hypertensive patients treated with the AIIRB losartan[34]. Furthermore, the RENAAL trial showed[35] that progression to end-stage renal disease in hypertensive diabetics could be delayed by losartan, compared to antihypertensive treatments that do not inhibit RAS. These beneficial effects of RAS blockers have been confirmed by other investigators[36,37]. All in all, these findings demonstrate that antagonizing the RAS is a useful tactic to prevent endothelial dysfunction, inflammation, and atherosclerosis. It remains for us to learn whether the different approaches to inhibit the RAS are all effective.

## Blocking of the AT1 Receptor

In animal models, blockade of the Ang II receptor has consistently demonstrated diminished expression and diminished serum levels of inflammatory markers as well as diminished cellular infiltration. In hypercolesterolemic normotensive monkeys, AIIRB causes regression of atherosclerotic plaques and decreased serum levels of inflammatory markers[32].

These results have been validated by studies in humans showing that the Ang II–induced stimulation of a number of inflammatory markers (ICAM-1) and many others can be reversed by losartan. In particular, losartan lowers serum MCP-1, a monocyte chemoattractant[38], and serum PGF2α, an indicator of monocyte COX-2 activity[39]. In contrast, treatment with atenolol does not prevent the angiotensin-induced rise in ICAM-1, MCP-1, or PGF2$\alpha$.

Several clinical studies have shown the beneficial effects of AIIRB. The Losartan Intervention for Endpoint Reduction (LIFE) Trial[40] showed that losartan decreases LVH in hypertensive patients compared to atenolol. In this study, the beneficial effects of losartan were unrelated to blood pressure levels, suggesting nonhemodynamic actions on the myocardium. In addition to the previously mentioned RENAAL study using losartan[35], the IRMA II[41] and the IDNT study[42] assessed the effects of ibersartan on diabetic renal disease. They also showed benefits above and beyond blood pressure. All the results were consistent with previous finding in humans' studies reporting that blockade of Ang II receptors decreased proteinuria more efficiently than other treatments, even though blood pressure was controlled at similar levels.

All in all, these studies support the notion that in hypertension, AIIRB inhibits the synthesis of a number of inflammatory molecules. Consistent with this notion, other studies have also shown that losartan slows down vascular disease progression and decreases platelet adhesion compared to patients not treated with an AIIRB[43].

## Inhibition of Angiotensin-Converting Enzyme

No doubt, the antihypertensive effects of ACE-I can reverse the direct effects of blood pressure on cytokine levels[12]. However, ACE-I have also shown anti-inflammatory effects in normotensive animal models. For example, quinapril decreases the expression of NF-κB, as well as IL-8 and monocyte chemoattractant protein-1 in a rabbit model of atherosclerosis[44]. Other animal models of atherosclerosis and vascular injury have shown benefits from ACE inhibition[45]. Hence, identification of inflammatory markers could be of help to recognize those patients that could benefit from the non-blood-pressure–related effects of ACE-I.

One of these Ang II–mediated markers of inflammation is the NF-κB transcribed in human monocytes, human vascular smooth muscle cells, and human endothelial cells. Ang II increases the production of vascular cell adhesion molecule-1[29], MCP-1[30], and IL-6[31]. All of these molecules are involved in the development of tissue inflammation in the kidney and in the cardiovascular system and all are inhibited by Ang II blockade.

These findings indicate that ACE-I is a useful tool to prevent endothelial dysfunction, inflammation, and atherosclerosis.

Moreover, ACE-I have unequivocal protective effects in patients with left ventricular dysfunction that are unrelated to hypertension control[37]. In this and in other populations at risk of vascular disease, the beneficial effects of ACE-I have been out of proportion to the degree of blood pressure lowering. In fact, in the HOPE trial, the benefit of the ACE-I ramipril was roughly three times greater than expected from blood pressure reduction alone[46]. These nonhemodynamic effects of ACE-I could result from anti-inflammatory actions.

## Blockade of Aldosterone Receptor

Aldosterone is a direct mediator of inflammation[47]. In addition, aldosterone can induce endothelial dysfunction by increasing Ang II through up-regulation of ACE expression[48]. Aldosterone stimulates collagen gene expression and synthesis via MR-mediated ERK1/2 activation in renal fibroblasts. This effect may be responsible for the progression of aldosterone-induced tubulointerstitial fibrosis[49].

Many of these aldosterone-related effects can be reversed by specific antagonists as shown both in experimental models. For instance, in a rabbit atherosclerotic model, eplerenone (an aldosterone receptor blocker) enhanced the endothelium-dependent relaxation[50], inhibited the development of atherosclerosis, and suppressed serum and macrophage oxidative stress[51]. Moreover, in hypercholesterolemic monkeys, 9 months of treatment with eplerenone suppressed MCP-1 and malondialdehyde-modified LDL. In the eplerenone-treated monkeys, the ratio of intimal volume to total volume was lower, and the acethylcholine-induced vasorelaxation was significantly enhanced. Of interest, eplerenone decreased plasma ACE to normal levels.

In humans, the RALES trial showed a significant increment in survival in chronic heart failure patients using spironolactone in addition to standard therapy, including diuretics and ACE-I. It has been shown that ACE-I only transiently suppress aldosterone production, and the RALES results suggest that aldosterone plays a significant independent role in cardiovascular disease[52].

In summary, the blockade of the rennin-angiotensin-aldosterone system at any step causes anti-inflammatory effects. The basic mechanism involves inactivation of the endothelium, and further cellular migration to the vascular, renal, or myocardial tissue is prevented; these effects are independent of changes of blood pressure.

## Blockade of the Beta Adrenergic Receptors

The data supporting the beneficial effects of beta-adrenergic receptor blockers in hypertension are less clear, but the antioxidative effect in myocardium is well studied. Several experimental studies have shown that catecholamines induce oxidative stress in the heart. In effect, isoproterenol induces lipid peroxidation and norepinephrine generates hydroxyl free radicals in animal hearts[53,54]. Therefore, beta-blocker therapy may prevent oxidative stress in patients with heart failure. Actually, Kukin et al.[55] reported that beta blockers, such as carvedilol and metoprolol, reduced plasma thiobarbituric acid–reactive substances, a marker of lipid peroxidation, in patients with heart failure.

Carvedilol is known not only as a vasodilating beta blocker, but also as an antioxidant. It also inhibits Fe2-initiated lipid peroxidation and partially prevents hydroxyl radical–induced cardiac contractile dysfunction, while propranolol does not[56]. A recent study revealed that carvedilol inhibits ROS generation by leukocytes[57]. These mechanisms may contribute to the reduction of 4-hydroxy-2-nonenal–modified protein, which is a major lipid peroxidation product, in human failing hearts.

## Evidence from Statins

The 3-hydroxy-3-methylgutaryl-CoA reductase inhibitors (statins) are potent inhibitors of cholesterol biosynthesis and have been shown to reduce ischemic events in numerous at-risk populations with varying degrees of cholesterol elevation. These agents slow the progression and foster the regression of atherosclerosis, resulting in an improvement of cardiovascular outcomes in humans with elevated and normal serum cholesterol levels[58].

Unfortunately, the results in hypertensive animal models have been somewhat ambiguous regarding renoprotection. Statins seem to decrease blood pressure, thereby limiting proteinuria and decreasing the expression of inflammatory markers. This anti-inflammatory response was observed by our laboratory in hypercholesterolemic rats. In effect, treatment with atorvastatin prevented endothelial dysfunction, proteinuria, and cholesterol-induced intrarenal proliferation of leukocytes[59]. In agreement with these results, other investigators have shown that pravastatin improves coronary endothelial function in cynomolgus monkeys treated with an atherogenic diet for 2 years, independent of the serum lipoprotein concentrations[60]. Similar results have been reported in experimental porcine hypercholesterolemia[61].

Clinical studies are consistent with the animal studies. A recent small randomized trial showed that atorvastatin significantly slowed renal function loss in people with proteinuric renal disease[62]. The Heart Protection Study showed a beneficial effect of simvastatin on the rate of kidney function loss[63]. In contrast, a large randomized study in renal transplant recipients found no evidence that fluvastatin improved graft survival[64], although it is unclear whether these findings are applicable to native kidney disease, in which the causes and mechanisms of progressive renal loss might differ substantially[65]. A subgroup analysis of the CARE trial showed that pravastatin reduced the rate of kidney function loss, especially in those patients with baseline glomerular filtration rate ≤ 40 ml/min per 1.73 $m^2$[66]. Recent work in experimental and human renal disease demonstrates that statins reduce urinary protein excretion[67]. Because proteinuria is associated with more rapid rates of kidney function loss, this finding suggests that individuals with detectable urinary protein might be more likely to derive renal benefit from statins. Altogether, the evidence suggests that direct anti-inflammatory effects may be the primary mode of action of statins. These lipid-independent or “pleiotropic” effects on renal and cardiovascular diseases include restoration of endothelial function, stabilization of atherosclerotic plaques, and decrease in oxidative stress–dependent vascular inflammation[68]. These pleiotropic effects of statins may function through several mechanisms. For instance, statins can block the synthesis of various isoprenoid intermediates that serve as lipid attachments for the post-translational modification of a variety of intracellular signaling proteins[69]. In addition, statins may reduce inflammation by: (a) up-regulation of eNOS expression and activity, (b) decreased ET-1 expression, (c) down-regulation of Ang II receptor subtype 1 (AT1) expression, and (d) inhibition of NAD(P)H oxidase activity[70,71,72].

One important mechanism by which statins exert anti-inflammatory actions and thus improve endothelial function is the enhancement of vasodilator (NO and prostacyclin) and attenuation of vasoconstrictor (Ang II and ET-1) activity in the vascular wall. *In vitro*, statins increase NO bioavailability by rising eNOS expression, mainly through post-transcriptional mechanisms that entail the blocking of geranylgeranylation of the small GTP-binding protein Rho through to inhibition of the biosynthesis of GGPP[72,73,74]. Similarly, simvastatin has been shown to up-regulate eNOS activity by activation of the serine/threonine kinase Akt (also named protein kinase B) in endothelial cells. The resulting phosphorylation of eNOS increases its activity and enhances NO production[75]. Atorvastatin also down-regulates the cellular expression of caveolin-1, which acts as an inhibitor of eNOS activation by forming an heterocomplex with this enzyme[76]. While other mechanisms have been described[77], the potential clinical meaning of all these observations is exceedingly promising, as the prophylactic treatment of normocholesterolemic mice with statins increases cerebral blood flow, reduces cerebral infarct size, improves neurological function[78], and improves myocardial function in animal models subjected to global myocardial ischemia and reperfusion[79,80] via a NO-mediated mechanism. Taken together, there is now clear evidence from experimental and clinical studies that increasing endothelial NO release is a common property of statins, independent from their impact on cholesterol synthesis.

Another mechanism by which statins may prevent inflammation is by decreasing ET-1 synthesis. This may result indirectly by increasing NO bioavailability, as described above. On the other hand, it has been shown that both atorvastatin and simvastatin reduce the expression of pre-pro-endothelin-1 mRNA and the synthesis of ET-1 in a concentration- and time-dependent manner in bovine aortic endothelial cells[68] and restored the response to ET-1–normalized endothelial function and vascular response to ET-1 in Dahl salt-sensitive rats[80]. Finally, these effects on Ang II, ET-1, and NO induce less adhesiveness, and prevent migration and inflammation in the interstitium by decreasing the synthesis of several adhesion proteins, such as LTB4, LFA-1, P-Selectin, ICAM-1, IL-6, IL-1β, MCP-1, PAF, and TGF-β1.

## SUMMARY

In recent years, much attention has been paid to the slow inflammatory process observed in hypertension; in particular this mediated by Ang II, endothelin, and other autacoids. However, the notion of an inflammatory process underlying cardiovascular disease was suspected in the 1950s. Karmen et al.[81] reported that enzyme release from necrotic cardiac myocytes could be detected in the serum and could help in the diagnosis of myocardial infarction. The ensuing years witnessed a progressive improvement in the cardiovascular tissue specificity of biochemical markers and a corresponding enhancement in the clinical sensitivity and specificity of their use.

Nowadays, hs-CRP, TNF-α, IL-6, ICAM-1, MCP-1, and others are important markers of the inflammatory response in hypertension. The expression of all these molecules is regulated by interacting intermediate inciters such as Ang II, ET-1, NO, aldosterone, etc. That is why ACE-I, AIIRB, statins, and aldosterone blockers reduce the markers of the slow inflammatory response in the cardiovascular system.

The beneficial effect of lowering these inflammatory markers is reflected in improved outcomes in several clinical trials. Nevertheless, for some of these drugs with antihypertensive actions, the improved outcomes may be due, at least in part, to the hemodynamic effect of lowering blood pressure.

For the near future, there is growing evidence that a multimarker strategy, employing biological biomarkers, will likely help in the assessment of patients with different forms of cardiovascular disease.

## ACKNOWLEDGMENTS

The work performed in the authors' laboratories is funded by the J. Robert Cade Foundation and the National Council of Scientific and Technical Research (CONICET).
